# The Effects of Bilirubin and Lumirubin on Metabolic and Oxidative Stress Markers

**DOI:** 10.3389/fphar.2021.567001

**Published:** 2021-03-04

**Authors:** Aleš Dvořák, Kateřina Pospíšilová, Kateřina Žížalová, Nikola Capková, Lucie Muchová, Marek Vecka, Nikola Vrzáčková, Jana Křížová, Jaroslav Zelenka, Libor Vítek

**Affiliations:** ^1^ Institute of Medical Biochemistry and Laboratory Diagnostics, Faculty General Hospital and 1^st^ Faculty of Medicine, Charles University, Prague, Czechia; ^2^ 4^th^ Department of Internal Medicine, Faculty General Hospital and 1^st^ Faculty of Medicine, Charles University, Prague, Czechia; ^3^ Department of Biochemistry and Microbiology, University of Chemistry and Technology, Prague, Czechia; ^4^ Department of Paediatrics and Inherited Metabolic Disorders, 1^st^ Faculty of Medicine, Charles University, Prague, Czechia

**Keywords:** antioxidant, bilirubin, cell respiration, lumirubin, intracellular metabolite

## Abstract

For severe unconjugated hyperbilirubinemia the gold standard treatment is phototherapy with blue-green light, producing more polar photo-oxidation products, believed to be non-toxic. The aim of the present study was to compare the effects of bilirubin (BR) and lumirubin (LR), the major BR photo-oxidation product, on metabolic and oxidative stress markers. The biological activities of these pigments were investigated on several human and murine cell lines, with the focus on mitochondrial respiration, substrate metabolism, reactive oxygen species production, and the overall effects on cell viability. Compared to BR, LR was found to be much less toxic, while still maintaining a similar antioxidant capacity in the serum as well as suppressing activity leading to mitochondrial superoxide production. Nevertheless, due to its lower lipophilicity, LR was less efficient in preventing lipoperoxidation. The cytotoxicity of BR was affected by the cellular glycolytic reserve, most compromised in human hepatoblastoma HepG2 cells. The observed effects were correlated with changes in the production of tricarboxylic acid cycle metabolites. Both BR and LR modulated expression of PPARα downstream effectors involved in lipid and glucose metabolism. Proinflammatory effects of BR, evidenced by increased expression of TNFα upon exposure to bacterial lipopolysaccharide, were observed in murine macrophage-like RAW 264.7 cells. Collectively, these data point to the biological effects of BR and its photo-oxidation products, which might have clinical relevance in phototherapy-treated hyperbilirubinemic neonates and adult patients.

## Introduction

**FIGURE 1:**
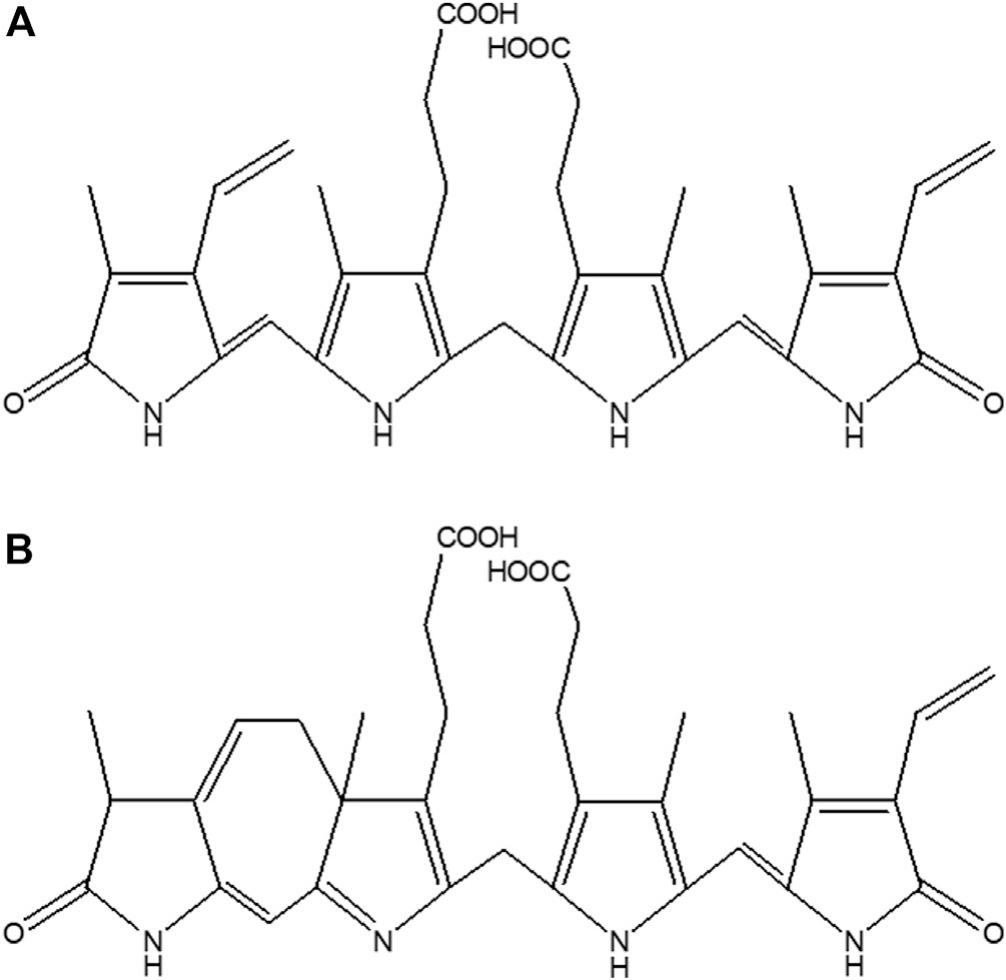
Structure of **(A)** BR, and **(B)** LR.

Bilirubin (BR) (Figure 1A), the major product of the heme catabolic pathway in the intravascular compartment, has been identified as a molecule of unique biological significance. Whereas in the past BR was merely considered as waste and a potentially neurotoxic product of heme catabolism, experimental as well as clinical research over the recent decades has convincingly proven its important antioxidant, anti-inflammatory, and other positive biological effects (Wagner et al., 2015).

Thus, mild hyperbilirubinemia exhibits protective effects against various chronic diseases mediated by increased oxidative stress (Wagner et al., 2015). Long-term, mildly elevated BR concentrations protect mitochondria and the respiratory chain, with a concomitant decrease of reactive oxygen species (ROS) and pro-inflammatory cytokine production (Zelenka et al., 2016); these observations are consistent with our previous *in vitro* and *in vivo* data, further demonstrating the anti-inflammatory effects of BR (Valaskova et al., 2019). This data are also in line with recent observations on beneficial effects of BR on metabolic pathways implicated in pathogenesis of diabetes, metabolic syndrome and obesity (Stec et al., 2016; Hinds and Stec, 2019) proposing BR as a signaling molecule with “real” endocrine activities (Hinds and Stec, 2018; Vitek, 2020). These pathways include those activated by PPARα (Stec et al., 2016; Hinds and Stec, 2018; Hinds and Stec, 2019), although signaling pathways activated by other nuclear as well as cytoplasmic receptors are likely to contribute as well (Vitek, 2020).

On the other hand, BR virtually behaves as a yin/yang molecule, being beneficial when only mildly elevated, while harmful when overcoming a safe threshold (Wagner et al., 2015). In fact, due to its lipophilic nature BR binds to myelin-rich membranes, insulating neurons and consequently affecting their function (Watchko and Tiribelli, 2013). BR-induced changes in the CNS are multifactorial, with extensive impacts on the brain compartment, inflammatory status, morphology, and are followed by cognitive dysfunction (Dal Ben et al., 2017). In cultured rat neurons, BR causes DNA fragmentation (Grojean et al., 2000), decreases respiration, changes both membrane potential and permeability, releases cytochrome c from the mitochondria to cytosol, and initiates apoptosis *via* caspase 3 (Rodrigues et al., 2002). Similar effects, including suppression of respiration followed by mitochondrial swelling leading to apoptosis, were observed in other cell types as well (Mustafa et al., 1969; Noir et al., 1972; Almeida and Rezende, 1981).

To some extent, neonatal jaundice is believed to play a protective role against increased oxidative stress (Hegyi et al., 1994; Shekeeb et al., 2008; Hansen et al., 2018). However, a serum BR concentration above 340 μmol/L is potentially dangerous for neonates with a high risk of BR neurotoxicity (Hyperbilirubinemia, 2004). The gold standard treatment for severe newborn jaundice is phototherapy (PT), which is generally considered a safe therapeutic method (AAP Subcommittee on Hyperbilirubinemia, 2004). During blue-green light PT (400–520 nm), BR is converted to photo-oxidation products that can be more effectively excreted into the urine and/or bile (McDonagh and Palma, 1980; Onishi et al., 1981; Lightner et al., 1984; Maisels and McDonagh, 2008). These products include, among others, BR photoisomers *Z,E*-BR IXα, *E,Z*-BR IXα, *Z*-lumirubin (LR) (Figure 1B), as well as monopyrrolic (BOX A and BOX B), dipyrrolic (propentdyopents) and tripyrrolic (biopyrrin A and biopyrrin B) oxidation products (Jašprová et al., 2018). Surprisingly, the biological properties of BR photoisomers and oxidation products are only poorly understood, primarily because of the absence of commercial standards for BR photoisomers.

Nevertheless, the metabolism of BR photoisomers seems to have clinical importance. Although PT is an effective, non-invasive, and relatively safe therapeutic method, accompanying potential side effects of the treatment are known in clinical practice, including: hypocalcemia (Khan et al., 2016), dehydration (Xiong et al., 2011), ileus (Raghavan et al., 2005), type 1 diabetes (Mcnamee et al., 2012), allergies (Safar and Elsary, 2020), increased incidence of cancer at advanced age (Auger et al., 2019), and even increased mortality of extremely low birth-weight infants (Arnold et al., 2014).

Hence, the aim of the present study was to investigate the biological properties of LR, the most abundant BR photoisomer, in order to account for some of the clinical observations in PT-treated neonates. So far, the intracellular metabolic impact of BR photoisomers has never been properly investigated, although our previous data suggest their biological importance (Jasprova et al., 2016; Jašprová et al., 2018). Therefore, we had aimed to study the *in vitro* effects of BR and LR on redox homeostasis and energy substrate metabolism.

## Materials and Methods

### Chemicals

All chemicals and cell culture reagents were obtained from Sigma-Aldrich (MO, United States) unless otherwise specified. Commercial BR was purified as previously described (McDonagh and Assisi, 1972) and diluted in DMSO or a stoichiometric concentration of bovine serum albumin (BSA) in PBS (Jašprová et al., 2018). BR stock solution (10 mmol/L in DMSO) was divided into aliquots and stored at −20°C to prevent degradation from repeated thawing. LR was isolated from the irradiated BR solution (BR was dissolved in a rabbit serum albumin solution) as previously described (Jasprova et al., 2020). For PPARα activity experiments, BR was dissolved using serum/albumin-free conditions (see below). The LR was dissolved in PBS with the concentrations measured spectrophotometrically (TECAN Infinite M200, Switzerland). LR stock solution (1 mmol/L) was divided into aliquots and stored at −20°C.

### Cell Cultures

Human neuroblastoma SH-SY5Y cells, hepatoblastoma HepG2 cells, murine macrophage-like RAW 264.7 cells, and fibroblast-like MRC5 cells from normal lung tissue were all purchased from the American Type Culture Collection (ATCC, VA, United States). HepG2, SH-SY5Y, and MRC5 were cultured in Minimum Essential Medium (MEM; Biosera, France) with 5 mmol/L glucose, and supplemented with both 2 mmol/L glutamine, 10% fetal bovine serum (FBS; Biosera), as well as with 1% non-essential amino acids (Biosera) in a normoxic CO_2_ chamber at 37°C in a humidified atmosphere. RAW 264.7 cells were cultured in Dulbecco’s Modified Eagle’s Medium (DMEM with 4,500 mg/L glucose, L-glutamine, and sodium bicarbonate) with 10% FBS.

The medium was replaced 24–48 h prior, starting the experiments with fresh medium containing LR and BR in a final concentration of 5, 25, or 50 μmol/L (control samples were treated by medium containing an adequate volume of LR/BR solvents; final concentration of DMSO = 0.5% v/v, concentration of BSA in FBS = 2.5 g/L (Soutar et al., 2019)). Thus, the respective concentrations of Bf (Bilirubin free, unbound, biologically active fraction of bilirubin) corresponded to non-toxic, borderline toxic and toxic concentrations, respectively (Roca et al., 2006; Zelenka et al., 2012). On the day of the experiment, the control cell culture reached a confluence of 80–90%.

### GC-MS Analysis of Intracellular Metabolites of the Tricarboxylic Acid Cycle

The cell samples (pellets washed with PBS) with an internal standard (IS, oxalate) were extracted with water/methanol/chloroform (1:1:2, v/v/v) and centrifuged at 1,000 × g for 10 min. The upper polar phase was transferred into a glass vial and lyophilized. The analytes were derivatized with pyridine/*N,O*-bis(trimethylsilyl)acetamide/chlorotrimethylsilane (8:4:2, v/v/v) at 65°C for 75 min. Derivatized samples were injected directly into a gas chromatograph - mass spectrometer (GC-MS, GC 6890N, MD 5973, Agilent Technologies, CA, United States) (Dvorak et al., 2017). The analyte amount was normalized to the IS and the cell count and calculated as a % of the control.

### High Resolution Respirometry

Cells pretreated for 24 h with BR/LR or their solvents were harvested and re-suspended in an adequate fresh medium (with BR/LR or their solvents) before measurement. The oxygen consumption of living cells was measured at 37°C using an Oxygraph-2k (Oroboros Instruments GmbH, Austria) in a 2 ml chamber in a MEM-based medium. The protein load reached roughly 3–15 mg/chamber, depending on the cell type. After air calibration and equilibration, the following inhibitors and uncoupler were used (SUIT-003 protocol): oligomycin (2.75 μmol/L), FCCP (6 μmol/L) (at least a 2-step titration), rotenone (0.2 μmol/L), and antimycin A (5 μmol/L). All respiratory parameters were analyzed in Datlab 7.4.0.4 software (Oroboros Instruments), and were expressed in pmol O_2_/s/mg protein (Smolkova et al., 2015).

The same procedure was used for experiments in serum-free medium. As recently reported, BR may affect *PPARα* expression and modify cell respiration and lipid metabolism (Gordon et al., 2020), but unsaturated fatty acids or their derivatives from FBS may compete with BR in active site (ligand-binding pocket) of specific nuclear receptors (Stec et al., 2016). Hence, in specific experiments FBS in medium was replaced by fatty acid free-BSA (to avoid the BR precipitation) 24 h before experiments. BSA was dissolved in PBS (43 g/L) and BSA solution was added to the FBS-free medium to a final concentration of BSA = 4.3 g/L (10% v/v).

### ATP Production

ATP amount was measured in cells exposed to a medium with glucose (normal phenotype) or galactose containing 25 μmol/L BR. Galactose in medium was selected to mimic starving leading to increase in the cell respiration, since decrease of ATP production by glycolysis must be compensated by increased oxidative phosphorylation.

The CellTiter-Glo Luminescent Cell Viability Assay (Promega Corporation, WI, United States) was used to determine ATP amount in the HepG2, MRC5 and SHSY5Y cells after BR treatment in substrate-specific conditions (galactose/glucose). For determination, MEM culture medium was replaced by DMEM-based medium 24 h before experiment (components were made to order and prepared by Institute of Molecular Genetics of the Czech Academy of Sciences). Experimental medium contained DMEM, 10% FBS, 1% Penicillin-Streptomycin, glutamine (2 mmol/L), glucose (5 mmol/L) or galactose (5 mmol/L), NaHCO_3_ (1.5 g/L) and BR (25 μmol/L) or respective solvent. To perform the assay, CellTiter-Glo reagent was prepared by reconstituting the lyophilized CellTiter-Glo substrate in the CellTiter-Glo buffer according to manufacturer´s instructions. Before the measurement the cells were washed with 100 µL of fresh medium to avoid artificial ATP detection from disrupted cells. Equivalent amount of the reagent was added to each sample and the signal was measured by a luminometer (Synergy/HT Microplate Reader, Biotek Instruments, Inc., VT, United Staes) at 22°C within 0, 5 and 10 min. The ATP concentration was normalized to the protein concentration measured immediately after luminometric analysis. The 96-well plate was divided in half and first part was used for luminometric method, whereas the second for the protein determination (treatment, seeding and time management were comparable). The wells were washed by PBS, and the lysis buffer (Buffer LYSIS LR, Biotechrabbit GmbH, Germany) was added, then the plate was vigorously shaken for 30 min on ice and the lysates were measured by Nanodrop (DS-11+ Spectrophotometer, DeNOVIX Inc., DE, United States) at 280 nm.

### Determination of Mitochondrial Superoxide Production

Superoxide production in live cells was detected using MitoSOX dye (Life Technologies, CA, United States). The cell suspension was stained for 15 min in a complete medium, then the sample was centrifuged (250 × *G,* 5 min); then the medium was removed and the pellet re-suspended in PBS. Fluorescence of the stained cells was measured by a flow cytometer (Mindray, BriCyte E6, China) every minute (0–15 min; 10,000 events/minute), and the change in proportion of superoxide-producing cells upon various treatments was monitored. The slope of fluorescence change, reflecting the production of superoxide in real time, was calculated. Rotenone (10 µM) was used as a positive control of the increased superoxide production in the treated cells (Zelenka et al., 2016).

### MTT Viability Test

The viability of cells exposed for 24 and 48 h to LR and BR was measured using the MTT test as previously described (Jašprová et al., 2018).

### Determination of Lipoperoxidation

The degree of lipoperoxidation was determined by a modified method, as previously described (Vreman et al., 1998). In brief, rat brain tissue (stored at -80°C until analysis) was diced, diluted 1:9 (w/w) in a phosphate buffer and sonicated. Next, 20 μl of this suspension was added to carbon monoxide (CO)-free vials together with 1 µL of LR or BR (with a final concentration range of 1–50 µM). The LR was dissolved in PBS or BSA, and the BR was dissolved in DMSO or BSA; while the pure solvents served as control samples. The Fenton reaction was initiated by a ferrous salt-ascorbate system injected across the airtight cap. Samples were incubated for 30 min at 37°C, and then the reaction was stopped by 10 µL of 60% (w/v) sulfosalicylic acid, then the samples were incubated for 30 min on ice. CO released into the vial headspace was quantified by gas chromatography (GC-RGD Peak Performer 1, Peak Laboratories, CA, United States), and reflected the lipoperoxidation rate.

### Determination of Serum Peroxyl Radical Scavenging Capacity

The serum peroxyl radical scavenging capacity (AOX, antioxidant capacity) was determined using a fluorimetric method, as the relative proportion of chain breaking antioxidant consumption present in the serum compared to that of Trolox (a reference and calibration antioxidant compound) (McDonagh and Assisi, 1972). The samples (human serum of healthy volunteer mixed with BR or LR; then LR, BR and Trolox dissolved in HSA, and also degraded LR dissolved in PBS measured after 6 and 24 h after dissolution were measured using a 96-well plate. Forty µL of dipyridamol (dissolved in DMSO and diluted to 2.5 μmol/l in PBS), 40 µL of sample (2.5, 5, 25 and 50 μmol/L BR/LR) and 20 µL of 2,2′-azobis(2-methylpropionamidine) dihydrochloride (100 mmol/L) were added into the reaction well and fluorescence (Ex. 415 nm, Em. 480 nm) was measured immediately. Total time of analysis was 2 h, fluorescence was measured every 2 min (plate was shaken periodically before each scan). For all samples, the time to fluorescence quenching was evaluated and was compared to respective control samples.

### Determination of the Glycolytic Reserve

The glycolytic reserve of the cells (SH-SY5Y, HepG2, and MRC5) was measured using a Seahorse XF24 Analyzer (Agilent Technologies), with a combination of two standard Agilent protocols (the Mito Stress and Glycolysis Stress tests). The combination of both protocols made it possible to check if the cells were respiring normally.

To improve cell adherence, the plate was coated with poly-L-lysine (100 μL of 0.01% solution per well). One day before the experiment, the cells were seeded by adding 100 μL of suspension (at density of 500,000 cells/mL for HepG2 cells, and 750,000 cells/mL for MRC-5 and SH-SY5Y). The plate with cells was left overnight in a humidified incubator supplemented with 5% CO_2_ at 37°C.

Assay medium preparation: D5030 DMEM assay medium was diluted in sterile water according to the manufacturer´s instructions, the pH was adjusted to 7.4 with NaOH, and then the solution was made to make the accurate volume. Then, the DMEM solution (99 ml) was mixed with 1 ml of L-glutamine (water stock solution, 200 mmol/L). The assay medium was heated to 37°C and the pH was checked. This medium was used for preparation of the stressor compounds. Stock solutions were prepared as 10x concentrated (glucose 50 mmol/L, oligomycin 20 μmol/L, FCCP 5 μmol/L, rotenone 20 μmol/L, antimycin A 10 μmol/L, 2-deoxyglucose 0.5 mol/L).

On the day of the experiment, the cell culture medium in a 24-well plate was removed and changed for 100 μL of the assay medium. The plate was placed in a humidified, temperature-controlled (37°C) incubator without CO_2_ atmosphere for 45–60 min for degassing, while the sensor cartridge was hydrated by loading of the stressor mix into every port A-D (A: 50 µL of glucose, B: 55 µL of oligomycin, C: 61 µL of FCCP, and D: 67 µL of 2-deoxyglucose, rotenone, and antimycin A).

The glycolytic capacity and reserve were evaluated as the difference of the extracellular acidification rate (ECAR) under conditions in the presence of oligomycin and glucose.

### Determination of Nitric Oxide Production

Production of nitric oxide (NO) was measured in the media by determination of nitrite concentration using the Griess reagent. Cells seeded in 96 wells were cultured in colorless medium and mixed with LR or BR (5 or 25 μmol/L), with or without lipopolysaccharide (LPS; 1 μg/ml), 24 h before measurement. Eighty µL of medium from each well were mixed with 80 µL of Griess solution (0.04 g/ml), and absorbance at 540 nm was measured by an iMark Microplate Reader.

### Quantitative Real-Time PCR

Total RNA was extracted using a GenUP Total RNA Kit (BiotechRabbit, Germany), and complementary DNA (cDNA) was synthesized with a High Capacity cDNA Reverse Transcription Kit (Applied Biosystems, CA, United States). Amplification of the target genes was performed on a ViiA 7 instrument (Applied Biosystems) in 10-μL reaction volumes, containing 4.5 μL of 10-fold diluted cDNA template from a completed reverse transcription reaction, TaqMan™ Fast Advanced Master Mix (Applied Biosystems) and TaqMan™ Gene Expression Assay (Mm00443260_g1 TNFα; Mm01545399_m1 HPRT, Hs00947536_m1 PPARα, Hs99999909_m1 human HPRT, Hs00354519_m1 CD36, Hs00173927_m1 FGF21, Hs00912671_m1 CPT1A, Hs01037712_m1 PDK4, Hs01101123_g1 ANGPTL4, Hs01005622_m1 FASN; Applied Biosystems). The temperature profile was: 2′ 50°C, 92°C 10′ 40x (1″ 95°C, 20″ 60°C). The relative quantification was made by the 2-ΔΔCt method with HPRT as a housekeeping gene.

For *TNFα* mRNA expression the murine macrophage-like RAW 264.7 cells were seeded in 6-well plates and incubated for 24 h with 5 and 25 μmol/L BR or LR. To assess the effect of LPS on *TNFα* expression, the cells were incubated for the last hour with 50 ng/ml of LPS. Then the cells were washed with PBS and collected in a lysis buffer (Buffer LYSIS LR, Biotechrabbit GmbH, Germany).

For *PPARα* and its downstream effector gene mRNA expressions (FASN, CPT1A, FGF21, PDK4, ANGPTL4 and CD36, (Rakhshandehroo et al., 2010)), the HepG2 cells were seeded in 12-well plates. The medium was replaced by the fresh serum-free medium for 24 h, then BR, LR or solvent was added. After 0.5, 1, 2, 4, 6 and 24 h, the cells were washed with PBS and collected as described above. To maintain serum and albumin-free conditions in these experiments, BR was dissolved in 0.1 M NaOH, neutralized by 0.1 M H_3_PO_4_ and buffered by PBS (0.01 M, pH 8.0, added in large excess). Final concentration of BR stock solution was 480 µM. LR was dissolved directly by PBS used for final step of BR solution preparation. Final concentration of BR/LR in medium was 25 µM.

### TNFα and FGF21 Protein Quantification

Concentrations of TNFα were measured in culture media (50 μL) removed from the murine cells RAW 264.7, incubated for 24 h with or without BR or LR (5 and 25 μmol/L), using a specific TNFα Mouse ELISA Kit (Thermo Fisher Scientific) according to the manufacturer’s instructions. For TNFα expression with LPS, 50 ng/ml of LPS were added into the medium 4 h before the end of a 24 h incubation.

Concentration of fibroblast growth factor (FGF) 21 was measured in culture media (50 μL) removed from the HepG2, incubated for 48 h in serum-free media with or without BR or LR 15 μmol/L), using a specific Quantikine ELISA Human FGF-21 Immunoassay kit (R&D Systems, United Kingdom) according to the manufacturer’s instructions. TNFα and FGF21 concentrations were measured in triplicates from individual wells on a microplate reader (TECAN Infinite M200). The concentrations were expressed in pg/mL. Due to increased toxicity of BR on HepG2 cells cultured in serum-free media, BR was used in concentration of 15 μmol/L and the concentrations of FGF21 in media were related to g of the cell lysate protein.

### Stability of LR and BR

The culture medium as well as the human serum of a healthy volunteer were mixed with LR and BR to a final concentration of 10 μmol/L. Both solutions (medium and serum) were incubated for 6 h in a CO_2_ incubator (humidified atmosphere, 37°C, 5% CO_2_). Spiked medium or serum (10 µL) were collected every h (0–6 h) and then immediately frozen at −80°C until the pre-analytical extraction. With the use of other solvents, the same procedure was performed for the evaluation of LR stability. The culture medium was used for hypoxic treatment as well (samples were collected in both normoxic and hypoxic conditions simultaneously). A special hypoxic box (OxyCycler GT4181CN, BioSpherix, NY, United States) within the CO_2_ incubator was used for the experiments. The atmosphere was controlled by OxyCycler software, with the low oxygen volume compensated for by nitrogen. Gases certified for tissue culture experiments were used (Air Products, Czech Republic).

### LC-MS/MS Analysis of LR and BR

Ten µL of liquid samples were spiked with 10 µL of IS (mesobilirubin, c = 5 μmol/L) (Frontier Scientific, UT, United States). The extraction was performed with 1 ml of basic methanol. The samples were vigorously shaken and vortexed, and then the mixture was centrifuged (16,000 × *G*, 30 min). Next, 100 µL of supernatant were pipetted into glass vials with an inert insert (suitable for LC-MS analysis), and 3 µL were directly injected into the LC-MS/MS.

LC-MS/MS analysis was performed using an HPLC (Dionex Ultimate 3000, Dionex Softron GmbH, Germany) equipped with a Poroshell 120 EC-C18 column (2.1 µm, 3.0 × 100 mm; Agilent Technologies). For the gradient elution, the phase was prepared by mixing 1 mmol/L of NH_4_F (Honeywell, International Inc., Morris Plains, NJ, United Staes) in water and methanol (Biosolve Chimie SARL, France). The analytes were detected by MS (TSQ Quantum Access Max with HESI-II probe, ThermoFisher Scientific, CA, United States) operated in a positive SRM mode (Jašprová et al., 2020).

### Detection of LR Oxidation Fragments

Samples of LR fragments (decay products) were obtained by the spontaneous degradation of LR dissolved in PBS (humidified atmosphere, 37°C, 5% CO_2_, overnight incubation). The fragments were determined by direct injection of deproteinated samples into the MS.

The MS was set to scan parent ions in the 100–1,050 m/z range, with a 1 s scanning period. The initial tuning of the heated HESI-II probe was performed according to a previously published paper of LR analysis (Jašprová et al., 2020). Further tuning of MS/MS transitions, derived from the most intensive parent ions, was performed by the combined infusion of the analytes (10 mg/L in the mobile phase, 20 μL/min) and the mobile phase (400 μL/min); the collision gas (Ar) pressure was set to 0.2 Pa.

Mono-, di-, and tri-pyrroles similarity was identified according to the described structures of known compounds (*e.g.*, monopyrrolic BOXes, propentdyopents, and biopyrrins). Fragmentation of tetrapyrroles as well as their ionization in MS were taken into account for in molecular weight estimations (assuming the charge number = 1).

### Statistical Analyses

The data are expressed as the mean ± SD. Depending on data normality, differences among variables were evaluated by the one-way ANOVA, Mann-Whitney Rank Sum test and Student unpaired *t*-test. Differences were considered statistically significant at *p* < 0.05. Statistical analyses were performed using Prism 8.0.1 software (GraphPad, CA, United States).

## Results

### Stability of LR and BR

**FIGURE 2:**
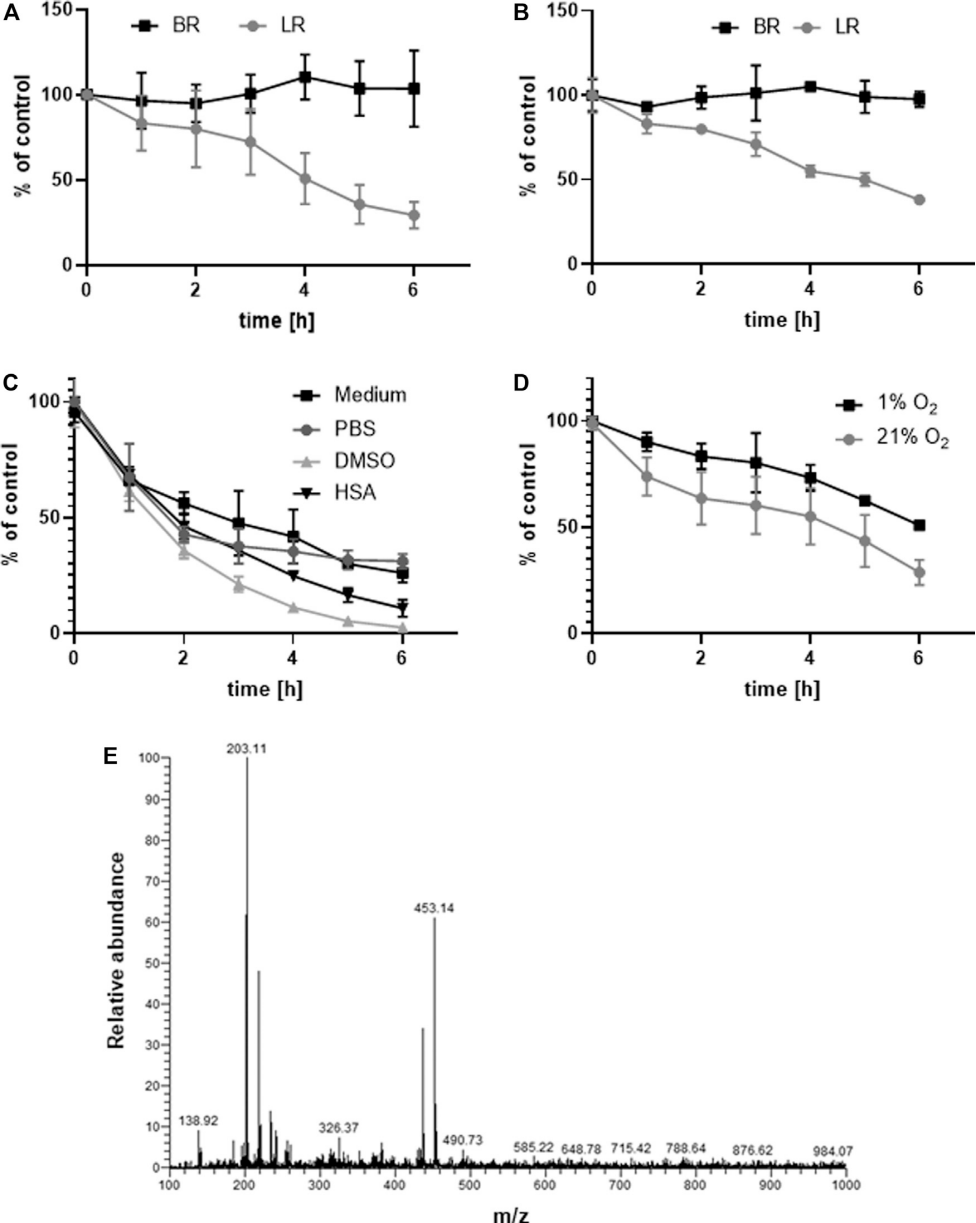
Stability of LR and BR. Stability of LR and BR (c_LR/BR_ = 10 μmol/L) was determined during 6 h in: **(A)** MEM medium spiked with LR and BR (n = 9); **(B)** human serum spiked with LR and BR (n = 9); **(C)** different solubilization media spiked with LR (n = 9); **(D)** MEM medium spiked with LR under hypoxic and normoxic conditions (n = 9); **(E)** ionizable degradation products of LR screened after 24 h of normoxic incubation in PBS (n = 1). BR, bilirubin; LR, lumirubin.

The stability of LR and BR in the relevant biological matrices were tested before any experiments with the biological samples. LR and BR concentrations were measured in samples of a standard medium (Figure 2A) and human serum (Figure 2B) spiked with LR and BR during 6-h incubation at 37°C in a CO_2_ atmosphere. The relatively fast degradation of LR was observed in both matrices. The half-life of LR was approximately 4 h; the remaining LR level after the 6 h-experiment was 29.3% and 38.2% in medium and serum, respectively. In contrast, BR was stable during the entire experiment. The half-life of LR was similar when using other solubilizing media (the rate of degradation in DMSO and PBS was comparable to that in MEM medium, even though the degradation curves were not identical; Figure 2C).

LR stability was also tested under different oxygen conditions (normoxia 21% O_2_ and hypoxia 1% O_2_) (Figure 2D). The presence of oxygen contributed to LR degradation beginning only 2 h after the start of incubation. The rate of degradation during the first 2 h was much faster under normoxic conditions (LR reduction: 8% per h in hypoxia, 17.5% per h in normoxia). After an additional 2–6 h, the same trend and rate of LR degradation was observed (LR reduction: 10% per h in hypoxia, 10.5% per h in normoxia) (Figure 2D). These results suggest that higher oxygen concentration can trigger LR degradation.

After 24 h of normoxic incubation of LR, degradation LR products were monitored with LC-MS/MS using a TIC scan between m/z 100–1,050. Two dominant (with m/z 203.1 and 453.1) and one less intensive (m/z 326.4) ions were observed in a MS scan of the PBS solution extract (Figure 2E). These are highly likely to be derived from mono-, di- and tripyrrolic structures (Lightner and Quistad, 1972; Yamaguchi et al., 1994; Seidel et al., 2014).

### The Effect of LR and BR on Cell Viability

**FIGURE 3:**
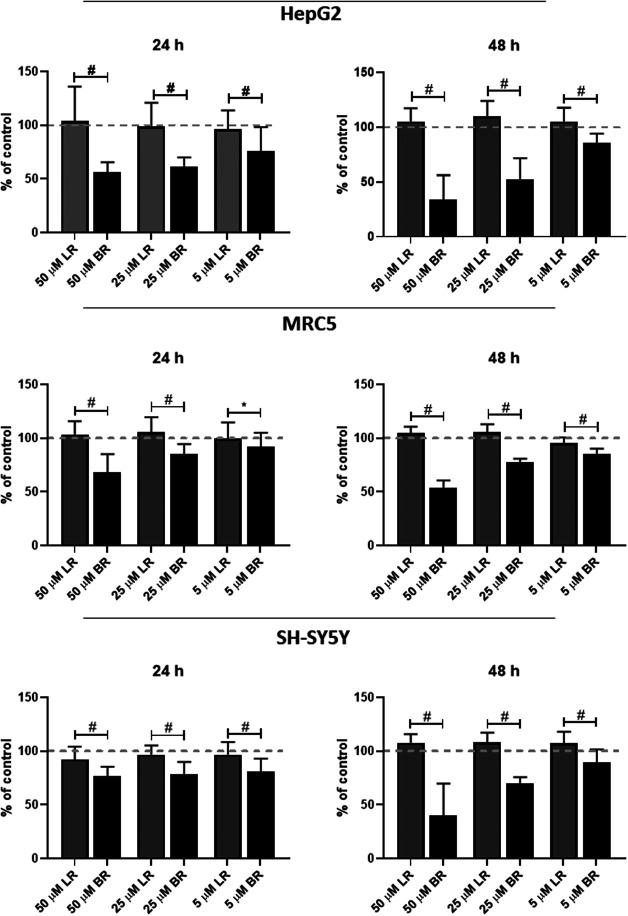
The effect of LR and BR on cell viability. Viability of cells (MRC5, HepG2, SH-SY5Y cells) was measured after 24 (left column) and 48 h (right column) by MTT test after treatment with lumirubin (LR) and bilirubin (BR) in different concentrations (5, 25, and 50 μmol/L). #, *p* < 0.001; *, *p* < 0.05. n = 48 for each experiment.

The effect of LR and BR on cell viability (SH-SY5Y neuroblastoma, HepG2 hepatoblastoma, and MRC5 lung fibroblasts cells) was measured by MTT test after 24 and 48 h-treatments with different concentrations of LR and BR. Whereas LR had no effect on the viability of any tested cells, BR concentrations negatively correlated with cell viability of all cell lines (Figure 3). A significant time- and concentration-dependent decreases in cell viability were observed after incubation with BR (*p* < 0.001 for almost all of the BR concentrations used, Figure 3); with HepG2 cells being the most sensitive to BR, most likely due to having the lowest glycolytic reserve (see below).

### Antioxidant Capacity of LR and BR

**FIGURE 4:**
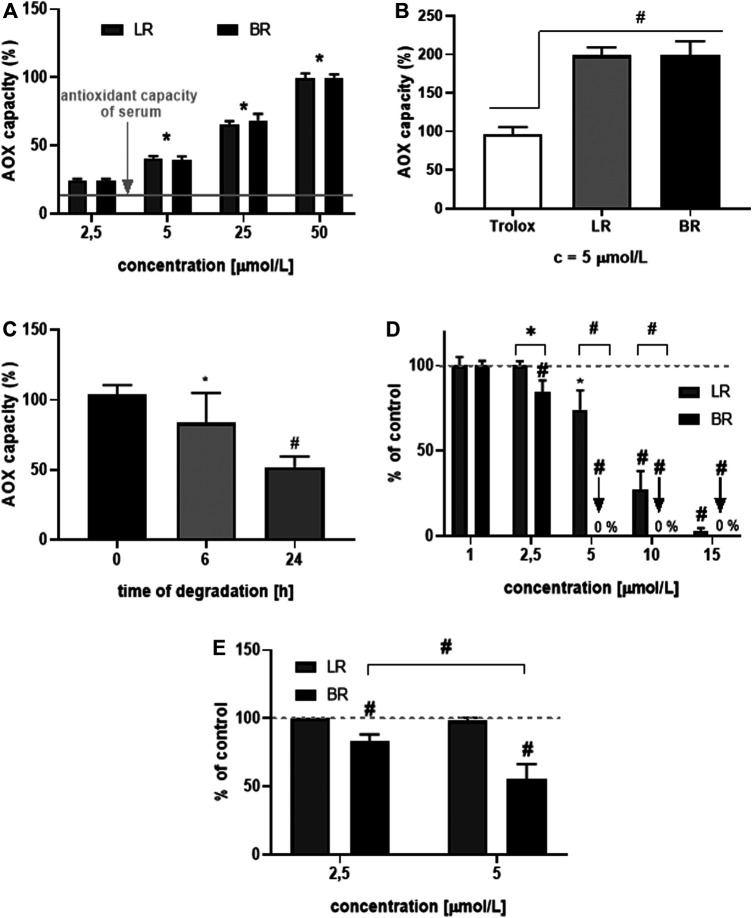
Antioxidant potential of LR and BR *in vitro*. **(A)** antioxidant capacity (AOX) in human serum spiked with LR and BR (n = 15); **(B)** AOX of HSA spiked with Trolox, LR and BR (n = 15); **(C)** AOX of LR in HSA after its spontaneous degradation (initial LR concentration = 25 μmol/L) (n = 6); **(D)** lipoperoxidation (LPX) of brain tissue after addition of LR (dissolved in PBS) and BR (dissolved in DMSO) (n = 8); **(E)** LPX of brain tissue after addition of LR and BR dissolved in BSA (n = 8). *, *p* < 0.05; #, *p* < 0.001. LR, lumirubin; BR, bilirubin.

In the next step, the AOX of LR and BR was tested in different biological matrices. First, we tested the capability of BR and LR to scavenge peroxyl radicals in human serum. BR and LR were added to the serum in increasing concentrations (2.5, 5, 25, and 50 μmol/L), which correlated well with the AOX of spiked serum (Figure 4A). Interestingly, LR had the same AOX as BR (Figure 4B) despite its degradation (Figure 4C). This observation was subsequently confirmed in the solution of human serum albumin (HSA) where both tetrapyrroles were compared to Trolox. The AOX of this vitamin E analogue was half that of BR and LR in the same concentration (5 μmol/L), respectively (Figure 4B). Because of the instability of LR (Figure 2C), the AOX of LR solutions with its spontaneous degradation was also tested. The AOX of LR solution decreased after 6 h to approximately 80% of the initial value (Figure 4C, *p* < 0.05), although the drop in LR concentrations reached 30% (Figure 2C). Even more interestingly, the decrease of AOX after 24 h reached approximately 50% of the initial value (*p* < 0.001, the data similar to that of Trolox from Figure 4B); although virtually no LR could be detected in the system (data not shown). This observation was suggestive of the antioxidant activity of the LR degradation products detected in our studies (Figure 2E).

Finally, the rate of lipoperoxidation (LPX) was measured in lipid-rich tissue (rat brain tissue). BR and LR were added to the matrix under different conditions. In the first set of experiments, LR was dissolved in PBS and BR in DMSO (Figure 4D); while in the other experiments, both tetrapyrroles were dissolved in BSA (Figure 4E). Surprisingly, a higher AOX of both compounds were observed in non-albumin solutions (Figures 4D,E). Compared to BR, LR was much less efficient in protecting lipid-rich cell homogenates from peroxidation, although still exerting a biologically relevant AOX (Figures 4D,E).

### The Effect of LR and BR on Superoxide Production

Production of mitochondrial superoxide was measured using a mitochondrial specific fluorescent probe MitoSOX, with two concentrations of LR and BR (5 and 25 μmol/L). Higher concentrations of BR (50 μmol/L) were too apoptotic, and cell debris interfered with determination of mitochondrial superoxide.

**FIGURE 5:**
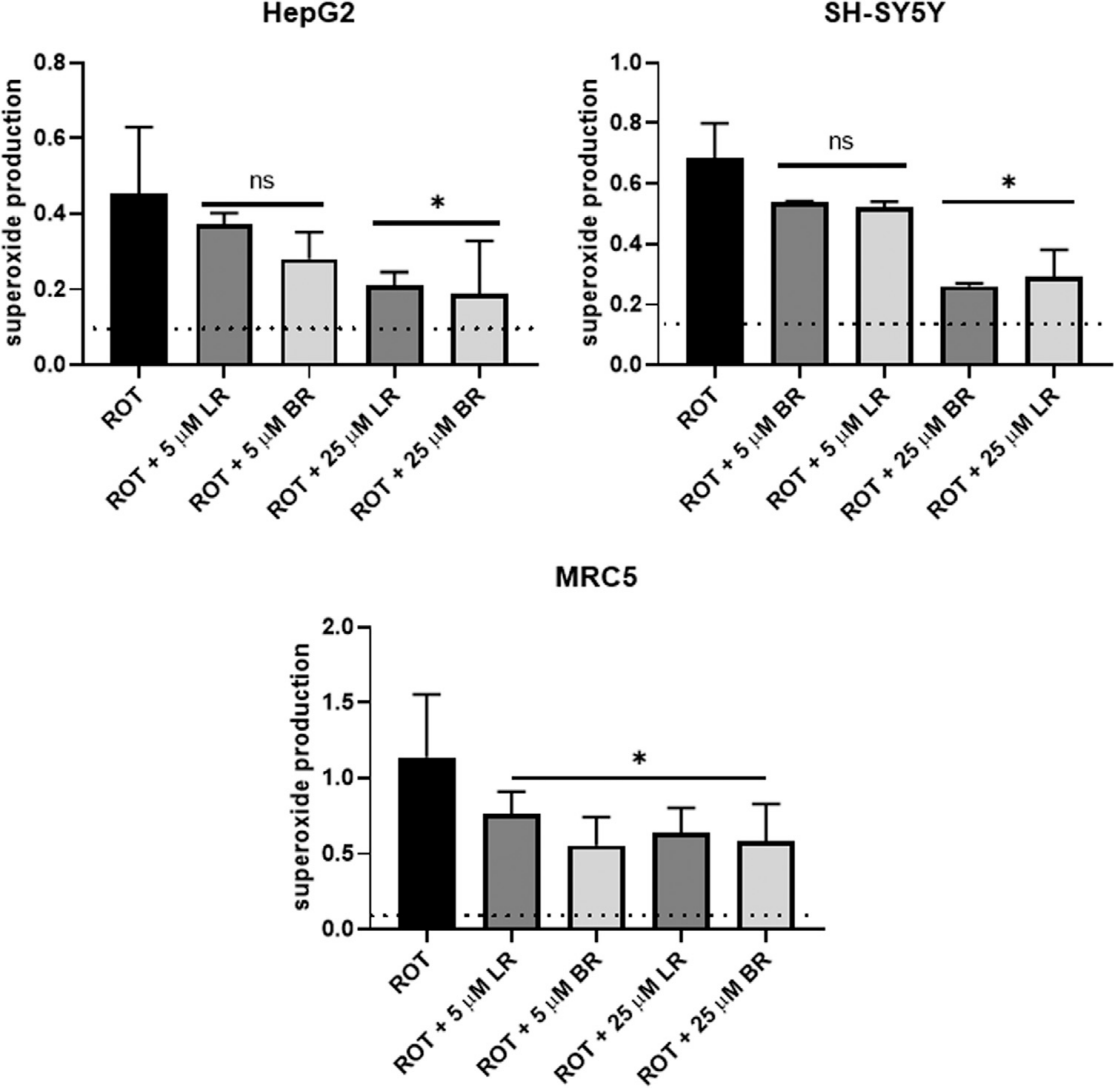
The effect of LR and BR on superoxide production. Superoxide production was determined by flow cytometry in live cells after overnight LR and BR treatment (5 and 25 μmol/L) from the detected slope. Rotenone (ROT) was used as a positive control of superoxide generation. Basal superoxide production (dashed lines) was detected in the cells without ROT treatment. *, *p* < 0.05; ns, not significant compared to ROT, n = 4 for each experiment CTRL, control cells without LR and BR treatment; BR, bilirubin; LR, lumirubin. Data expressed as the change in proportion of superoxide-producing cells upon various treatments.

After overnight pretreatment, both LR and BR were almost equally capable of the scavenging of mitochondrial superoxide in a concentration-dependent manner (Figure 5). In HepG2 and SH-SY5Y cells, only the higher concentrations of LR or BR caused a significant drop in superoxide production (Figures 5A,B); while in MRC5 cells, even the lower concentrations were significantly efficient (Figure 5C).

### The Effect of LR and BR on Mitochondrial Respiration

Since both pigments inhibited mitochondrial superoxide production, and previous studies had demonstrated an inhibitory role of BR on mitochondrial respiration (Mustafa et al., 1969; Noir et al., 1972; Almeida and Rezende, 1981; Rodrigues et al., 2002), we were thus interested in the effects of LR and BR on mitochondrial respiration in our cell models.

**FIGURE 6:**
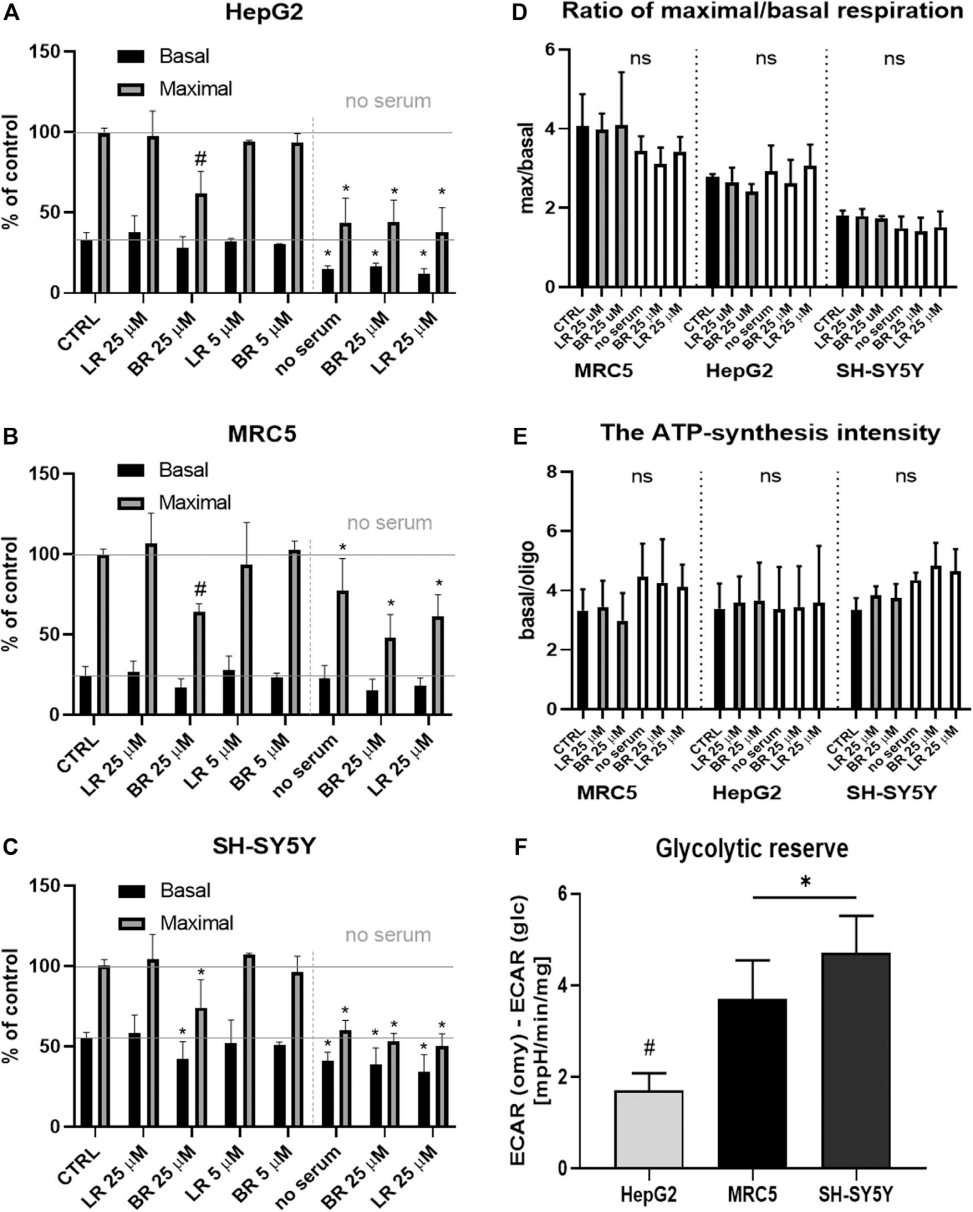
The effect of LR and BR on mitochondrial respiration. **(A–C)** Mitochondrial respiration of cells (HepG2, MRC5, SH-SY5Y) treated for 24 h with different concentrations (5 and 25 μmol/L) of LR and BR. Basal (endogenous) and maximal respiration were measured under presence of serum as well as serum-free conditions (only 25 μmol/L concentrations of BR/LR); **(D)** Ratio of maximal to basal respiration. White columns represent serum-free conditions; **(E)** ATP-synthesis intensity. White columns represent serum-free conditions; **(F)** Glycolytic reserve of tested cells. ECAR, extracellular acidification rate. *, *p* < 0.05; #, *p* < 0.001, *vs.* control; ns, not significant. n = 4 for each experiment A-E, n = 8 for experiments F.

Cell respiration was measured in all cell lines after overnight incubation with BR and LR in two different concentrations (5 and 25 μmol/L) (Figures 6A–C). Practically no changes in respiration were observed in any of the studied cells, except for the higher BR concentration (25 μmol/L), which decreased both basal as well as maximal respiration in all cell lines (Figures 6A–C). Substantial inhibition of respiration was observed under serum-free conditions, in particular in the cells of hepatic and cerebral origin; BR/LR treatment did not affect respiration in these specific conditions (Figures 6A–C).

The ratio of maximal to endogenous (basal) respiration, corresponding to the respiratory capacity (Brand and Nicholls, 2011), was different for each cell line: ± 4 for MRC5; ± 2.8 for HepG2; ± 1.8 for SH-SY5Y; with no significant changes between the controls and treated cells (Figure 6D). Serum-free conditions did not affect the ratio of maximal to basal respiration (Figure 6D).

The ATP-synthesis intensity was calculated as the ratio of basal (endogenous) respiration to respiration after inhibition of ATP-synthase with oligomycin. The values of the ATP-synthesis intensity under studied conditions did not differ (Figure 6E), suggesting that ATP-synthesis (or ATP-synthase involvement) is proportional to the reduction of maximal and basal respiration. Insignificant changes of both ratios between the control and cells treated with BR and LR indicated an overall depression of mitochondrial respiration with higher concentrations of BR, but not with LR.

**FIGURE 7:**
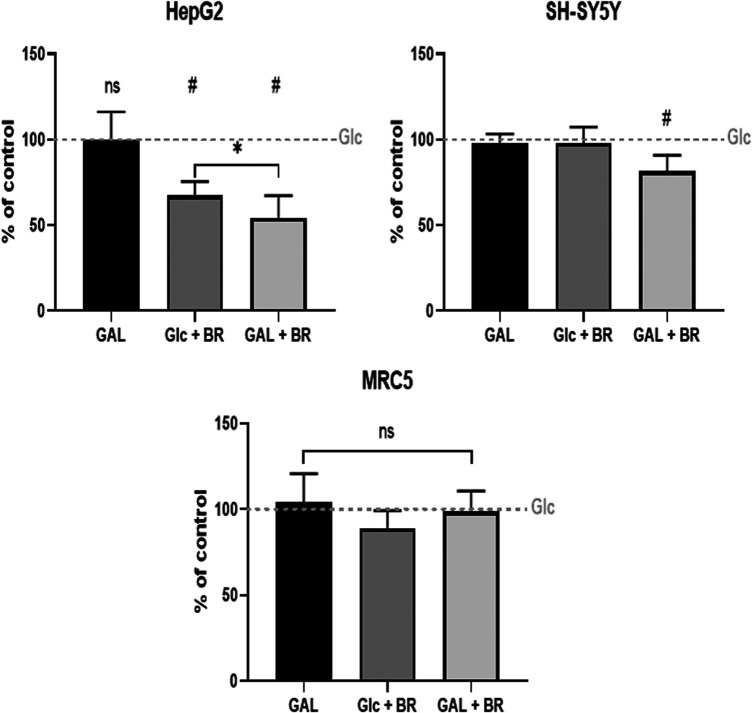
The effect of BR and LR on ATP production. Glc = glucose, 5 mmo/L; GAL = galactose, 5 mmol/L; BR = 25 μmol/L.

Finally, the glycolytic reserve of tested cell lines (without BR/LR treatment) was determined. The data demonstrate the highest glycolytic reserve in SH-SY5Y cells, whereas the lowest values were observed in HepG2 (Figure 6F). Interestingly, ATP production was not improved when glucose was exchanged for galactose; instead, it was further substantially decreased in the galactose medium in HepG2 cells, while only mild or no effects were observed in SH-SY5Y and MRC5 cells (Figure 7).

These results negatively correlated with the viability data, where the highest vulnerability was observed in HepG2 cells; contrasting with the lowest values observed in SH-SY5Y cells (Figure 3).

### The Effect of LR and BR on PPARα and its Downstream Effector Gene Expressions, and FGF21 Production

**FIGURE 8:**
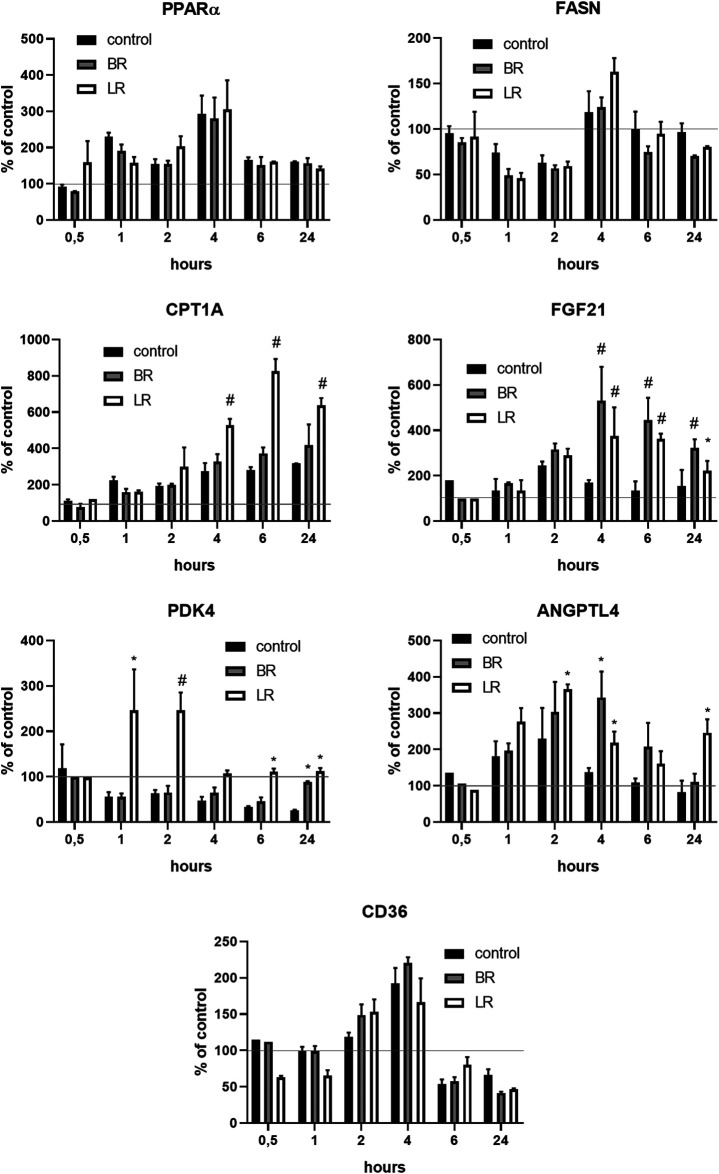
The effect of LR and BR on *PPARα* and its downstream effector gene expressions. The effect of LR and BR (25 μmol/L) on expression of *PPARα* and related genes involved in glucose and lipid metabolism was studied under serum-free conditions. The experiments were performed in 12-well plates; the medium was replaced by the fresh serum-free medium for 24 h, then BR, LR or solvent was added, and the cells were harvested after 0.5, 1, 2, 4, 6 and 24 h for gene expression analyses. FASN, gene coding for fatty acid synthase; CPT1, carnitine palmitoyltransferase 1; FGF21, fibroblast growth factor 21; PDK4, gene coding for mitochondrial pyruvate dehydrogenase lipoamide kinase isozyme 4; ANGPTL4, angiopoietin-like 4; CD36, cluster of differentiation 36, also known as platelet glycoprotein 4, fatty acid translocase, or scavenger receptor class B member 3. *, p < 0.05; #, p < 0.001, compared to control; N = 3.

**FIGURE 9:**
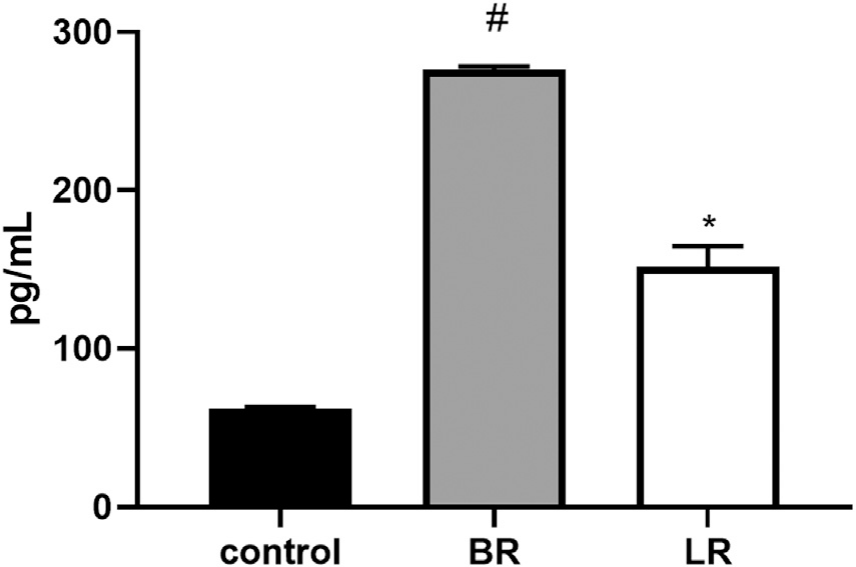
The effect of LR and BR on FGF21 protein production. Concentrations of FGF21 were measured in culture media removed from the HepG2, incubated for 48 h in serum-free media with or without BR or LR. Due to increased toxicity of BR on HepG2 cells cultured in serum-free media, BR was used in lower concentrations (15 μmol/L), and the concentrations of FGF21 in media were related to g of the cell lysate protein. *, *p* < 0.05; #, *p* < 0.001, compared to control; n = 3.

Since BR was recently demonstrated to improve metabolic functions in white adipose tissue *via* PPARα-activated mitochondrial metabolism (Gordon et al., 2020), we analyzed the effect of LR and BR on *PPARα* and its downstream effector gene expressions in HepG2 cells. While PPARα gene expression was not affected by exposure to both pigments, its downstream effector genes FGF21 and ANGPTL4 were significantly, time-dependently upregulated by both BR and LR. Interestingly, LR, but not BR significantly upregulated also *CPT1A* and *PDK4* gene expressions (Figure 8). Increased gene expression of FGF21 upon exposure to BR and LR was reflected by increased FGF21 production as demonstrated by significantly increased FGF21 concentrations in culture media (Figure 9).

### The Effect of LR and BR on Production of TCA Cycle Metabolites

Due to the impact of BR on mitochondrial metabolism, we next investigated the possible effects of LR and BR on the production of intracellular metabolites of the TCA cycle, known to not only affect energy balance but also to modulate multiple cellular functions (Martinez-Reyes and Chandel, 2020).

**FIGURE 10:**
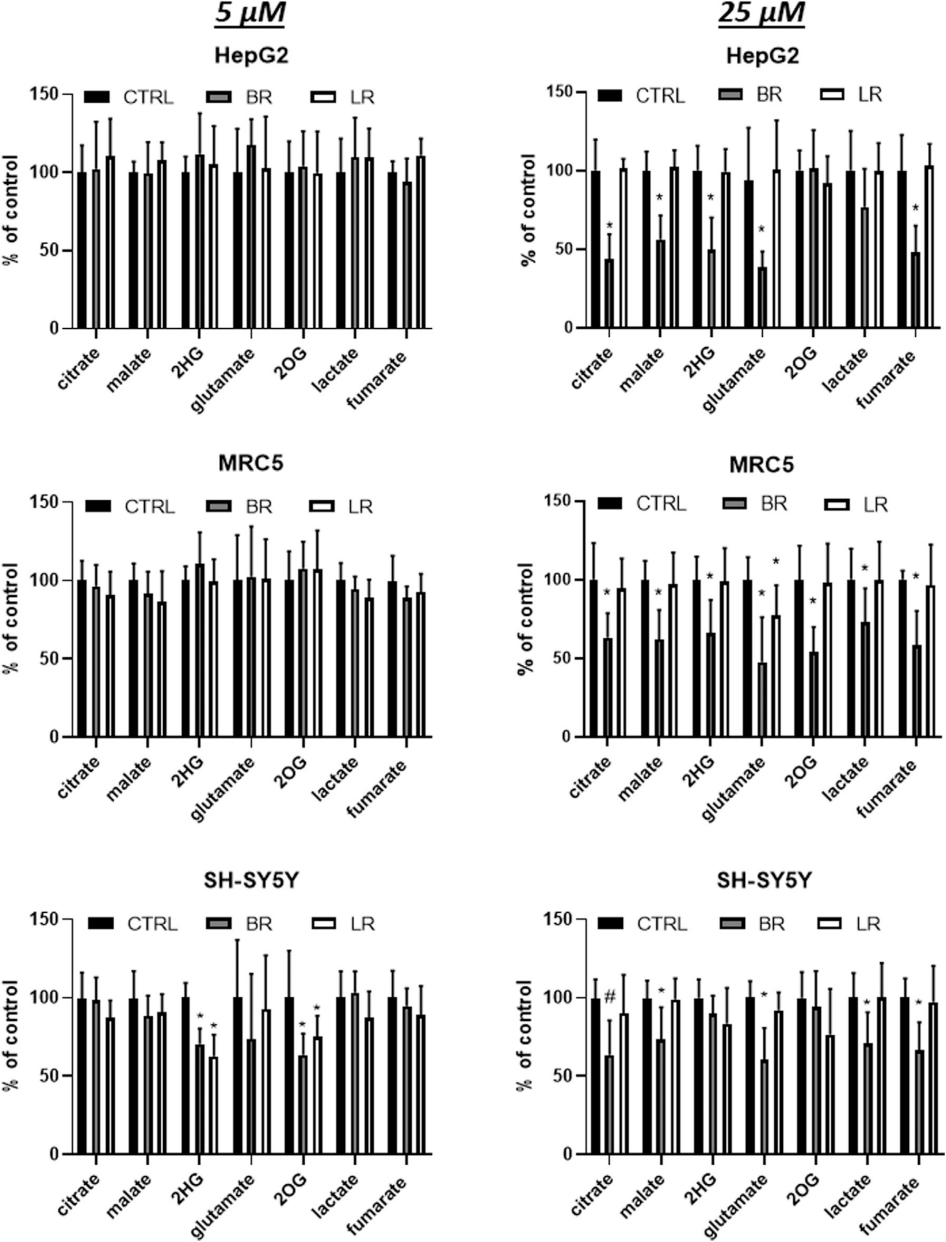
The effect of LR and BR on production of intracellular metabolites of TCA cycle. Concentrations of metabolic intermediates were measured in cells (HepG2, MRC5, SH-SY5Y) treated for 24 h with LR and BR (5 and 25 μmol/L). *, *p* < 0.05; #, *p* < 0.001. 2HG, 2-hydroxyglutarate; 2OG, 2-oxoglutarate; CTRL, control cells. n = 9 for each experiment.

Hence, the potential effects of LR and BR on the production of metabolic intermediates of the TCA cycle were measured in all investigated cell lines exposed to LR and BR. At lower concentrations (5 μmol/L), both compounds did not have any marked effect in MRC5 and HepG2 cells; while in SH-SY5Y cells the concentrations of 2-hydroxyglutarate and 2-oxoglutarate significantly decreased (*p* < 0.05) in the presence of both compounds (Figure 10).

A different response was observed with a 5x greater concentration of BR, with most metabolites significantly reduced in all cell lines; whereas virtually no effect was observed in cells exposed to LR (Figure 10).

### Anti-Inflammatory Effect of BR and LR

BR is a potent immunosuppressive compound (Jangi et al., 2013), but LR does not seem to act in the same manner (Jašprová et al., 2018). Thus, we were interested in the effects of BR and LR on TNFα expression as well as on NO production through inducible NO synthase (Xie et al., 1994).

**FIGURE 11:**
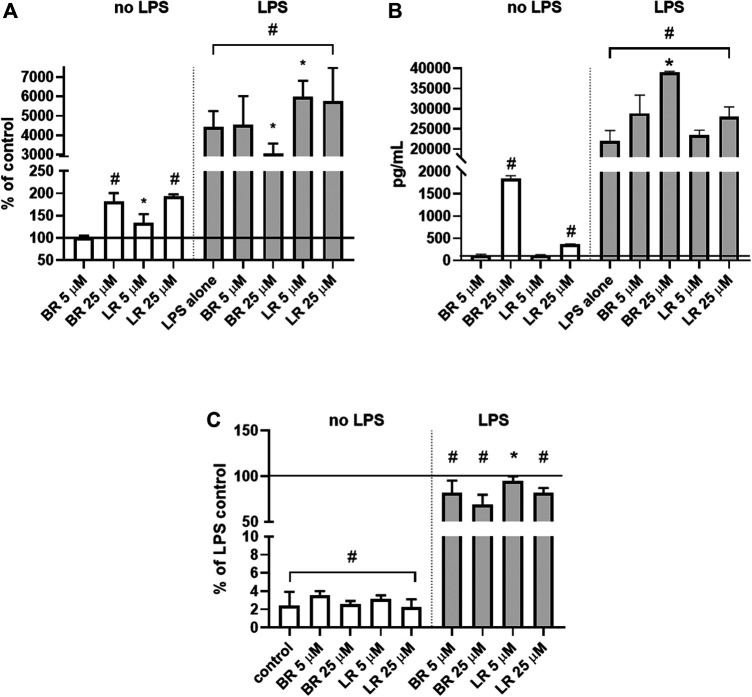
Anti-inflammatory effects of BR and LR in murine macrophage-like RAW 264.7 cells. **(A)** *TNFα* mRNA expression; (n = 6). **(B)** TNFα protein expression in culture media (n = 3). **(C)** NO production (n = 16). Murine macrophage-like RAW 264.7 cells exposed to LPS were treated for 24 h with different concentrations of LR and BR (LR/BR concentrations = 5 and 25 μmol/L). Horizontal line represents LPS control. *, *p* < 0.05; #, *p* < 0.001.

The anti-inflammatory effects of BR and LR were assessed in murine macrophage-like RAW 264.7 cells exposed to LPS. Basal *TNFα* expression was increased by both pigments (2-fold increase upon exposure to higher concentrations); with LR being effective in low concentrations (increase in *TNFα* expression by 40% at 5 μmol/L) (Figure 11A). When the cells were exposed to LPS, both pigments slightly, although significantly, modified *TNFα* expression (Figure 11A). More importantly, both pigments increased TNFα protein expression under unstimulated conditions (200- and 4-fold increase for BR and LR, respectively), and this effect persisted with almost 2-fold increase with 25 μmol/L concentration of BR (*p* < 0.05) (Figure 11B).

However, an increase in *TNFα* expression was not reflected by increased NO production (Figure 11C). On contrary, a decreased *TNFα* expression by higher BR concentration led to a decreased NO production, and a slight decrease in NO production was also observed with LR treatment despite its increasing effect on *TNFα* expression (Figure 11C).

## Discussion

During the last several decades BR has been increasingly recognized as an important bioactive molecule, with substantial toxic effects when it reached high concentrations within the human body (such as during the neonatal period). The gold standard treatment for severe neonatal jaundice is PT with blue-green light, generating more water-soluble BR photoisomers. Although considered safe, the biological properties of BR photoisomers and their oxidation products have not properly been investigated. However, the still scarce data obtained until now suggests some biological activity of these products (Jašprová et al., 2018), which may account for the reported clinical observations (Raghavan et al., 2005; Xiong et al., 2011; Mcnamee et al., 2012; Arnold et al., 2014; Khan et al., 2016; Auger et al., 2019; Safar and Elsary, 2020). In addition, practically no mechanistic studies have been performed to address these issues.

In our current study, we tried to compare and correlate the data on BR and LR cell toxicity with the parameters of mitochondrial metabolism and oxidative stress. As expected, compared to BR, LR was found to be much less toxic in all cell lines used including hepatic, fibroblast as well as neuronal models (Figure 3). The cytotoxicity of BR was affected by the cellular glycolytic reserve, which was most compromised in human hepatoblastoma HepG2 cells (Figure 6F). This data was consistent with the inhibitory effects of BR on mitochondrial respiration, and more importantly on the TCA cycle. In fact, BR in contrast to LR exhibited profound inhibitory effects toward TCA cycle metabolites, being tightly linked to oxidative phosphorylation (Martinez-Reyes and Chandel, 2020). BR inhibited the mitochondrial respiration of all tested cell lines of different origins. This is consistent with previous reports of the impact of BR on mitochondrial morphology and metabolism (Mustafa et al., 1969; Noir et al., 1972; Almeida and Rezende, 1981; Rodrigues et al., 2002), while LR did not have any serious harmful effect. Importantly, these inhibitory effects were demonstrated mostly in brain cells (Mustafa et al., 1969; Almeida and Rezende, 1981; Rodrigues et al., 2002), whereas in other tissues, such as liver or heart, the effects on mitochondrial metabolism were opposite, *i.e.* beneficial, especially in lower concentrations (Mustafa et al., 1967; Mustafa et al., 1969; Stumpf et al., 1985). In addition, beneficial effects of BR on mitochondrial function were also reported recently in adipocytes (Gordon et al., 2020). Hence, it seems that the effects of BR and its derivatives are complex, being cell-specific and dependent on concentration as well as other conditions. We were also able to confirm, consistent with a previous recent report (Gordon et al., 2019), potential metabolic activities of both BR and LR, as demonstrated by increased expressions of PPARα-dependent genes *FGF21* (reflected also by increased FGF21 protein production) and *ANGPTL4*, both involved in glucose and lipid metabolism. Interestingly, LR, but not BR significantly upregulated also the other important metabolic genes *CPT1A* and *PDK4* gene expressions, pointing to the possible metabolic importance of this BR photoproduct. Up-regulations of these PPARα-dependent genes became apparent under albumin-free conditions indicating that presence of albumin in the cell culture interfered with PPARα signaling mechanisms.

It is well known that deficiencies of respiratory complexes causes a drop in ATP production, with the appropriate metabolic consequences (Zielinski et al., 2016), including pseudo-hypoxic changes with hypoxia inducible factor (HIF)1α activation and pyruvate dehydrogenase inhibition (Kim et al., 2006), followed by an impairment of metabolic substrate utilization (Crewe et al., 2013; Kappler et al., 2019). Based on our data, it seems that inhibition of respiration may result in subsequent inhibition of anaplerotic pathways, and also to compensate for the undesirable and excessive production of NADH. This step may be crucial to avoid Krebs cycle overload associated with overwhelming redox stress (Koves et al., 2008).

An important finding of our study is the effect of LR on oxidative stress. LR exerted serum antioxidant capacity as well as mitochondrial superoxide production suppressing activity comparable to BR, which is known to be one of the most potent endogenous antioxidants (Stocker et al., 1987). Interestingly, a substantial antioxidant effect of LR was observed in our cell models despite its marked degradation, suggesting a marked ROS-scavenging activity of LR degradation products. Nevertheless, LR was much less efficient in preventing lipoperoxidation, most likely due to its lower lipophilicity.

Additionally, BR was found to behave as a pro-inflammatory molecule in the macrophage-like RAW 264.7 cells, while only mild and insignificant effect was observed for LR. This is in contrast with our previous study performed on different cell models of CNS origin (Jašprová et al., 2018) indicating substantial cell variability of BR/LR-induced pro-inflammatory effects. This observation may be linked to the BR-induced TCA cycle dysregulation known to affect inflammatory status, NO production, as well as post-translational acetylation (Williams and O'Neill, 2018). Interestingly, both treatments lead to a decrease in NO availability. Although inducible NO synthase activity is up-regulated by *TNFα*, and positive associations between *TNFα* and NO were reported in clinical settings (Soufli et al., 2016); BR is known to scavenge NO by forming N-nitroso derivatives (Barone et al., 2009), and the same might also be true for LR. In addition, BR inhibits inducible NO synthase (Zucker et al., 2015), and LR may also act in a similar manner.

One of the limitations of our study is that metabolic changes were only analyzed for LR, and not for the other specific BR photo-oxidation products (such as biopyrrins, propentdyopents, or monopyrrolic BOXes), which might have different biological impacts upon mitochondrial metabolism. In addition, the data from our *in vitro* experiments should be confirmed in *ex vivo,* animal and/or clinical studies.

Nevertheless, our data point to the biological effects of BR and its photo-oxidation products, which seem to have clinical relevance in phototherapy-treated hyperbilirubinemic neonates and adult patients.

## Data Availability

The raw data supporting the conclusions of this article will be made available by the authors, without undue reservation.

## Abbreviations:

AOX, antioxidant capacity; BSA, bovine serum albumin; BR, bilirubin; DMSO, dimethyl sulphoxide; HSA, human serum albumin; LR, lumirubin; PT, phototherapy
